# Collagen injections versus dry needling in the treatment of chronic supraspinatus tendinopathy: a randomized controlled trial

**DOI:** 10.3389/fsurg.2026.1771944

**Published:** 2026-03-13

**Authors:** Vincenzo Alessio Chirico, Giovanna Mazzuoccolo, Michela Saracco, Domiziano Tarantino, Carlo Ruosi, Bruno Corrado

**Affiliations:** Department of Public Health, University of Naples “Federico II”, Naples, Italy

**Keywords:** collagen, dry needling, injections, rotator cuff, tendinopathy

## Abstract

**Purpose:**

This randomized controlled study compares ultrasound-guided collagen injections (USG-CI) vs. ultrasound- guided dry needling (USG-DN) for treating chronic supraspinatus tendinopathy.

**Methods:**

Forty patients were randomly divided into two groups of 20 each. Group A received four weekly collagen injections, while Group B underwent two dry needling sessions four weeks apart. Both groups followed the same rehabilitation protocol. Outcomes were measured using the Constant-Murley Score (CMS), the Disabilities of the Arm, Shoulder and Hand questionnaire (DASH) questionnaire, and Ingwersen ultrasound score at baseline, 2 weeks, 1 month, and 3 months.

**Results:**

While no significant differences were found at 2 weeks, group A showed statistically significant improvements at 1 and 3 months compared to group B in both CMS and DASH scores. Ultrasound evaluation also revealed better structural improvement in group A. Both treatments improved pain and functionality, but USG-CI demonstrated a statistically significant advantage in clinical scores and tendon morphology at three months. The superiority of collagen injections may be attributed to their ability to stimulate tenocyte proliferation, endogenous collagen synthesis, and restoration of collagen fibers in damaged tendons. While USG-DN also showed therapeutic effects through induced bleeding and growth factor release, it was less effective overall.

**Conclusions:**

Our findings suggest that U S G - CI could be a safe and feasible treatment for supraspinatus tendinopathy. The future development of our study could be a predictive patient profiling, concomitant imaging and objective outcome measures to allow identifying patients most suitable for USG-CI. Our study has some limitations, so further studies are required to confirm these preliminary data.

## 1 Introduction

Tendinopathy is a chronic condition characterized by histological changes in the tendon structure, which lead to pain and reduced function. A healthy tendon is composed of extracellular matrix (ECM), mainly formed by a fibrillary network of parallel-aligned type I collagen fibers, and few cells (mostly tenocytes) aligned along the length of the collagen fibers (1, 2).

Loss of collagen structural organization, increase of type III collagen level, raised deposition of additional matrix proteins (proteoglycans and glycosaminoglycans), hypercellularity, and neovascularization are the main histological findings in tendinopathy (3). In the early phase of tendinopathy, type III collagen is overproduced and acts as a “patch” to protect the area of damage (4). Type III collagen is deposited haphazardly, contributing to the minor biomechanical strength and the irregular alignment seen microscopically in sick tendon (5, 6). In tendinopathy, the repair mechanism fails and consequent degeneration of the physiological tendon structure.

Supraspinatus (SSP) tendinopathy is the most common rotator cuff (RC) tendinopathy (7). The physio- pathological mechanisms of RC tendinopathy have been classically categorized as extrinsic, intrinsic or a combination of both. Extrinsic factors are defined as those causing compression of the RC tendons (due to the contact between tendons and bone structures), while intrinsic mechanisms are those originated within the tendon, usually because of overuse or overload.

Excessive tissue load remains the most substantial causative factor in the development of RC tendinopathy, and evidence suggests that intrinsic degeneration within the RC is the principal factor in the pathogenesis of RC tendinopathy and tears (8, 9).

First-line treatment of SSP tendinopathy is conservative; in about 80% of patients, non-surgical treatment relieves pain and improves function. Conservative therapies may include rest, activity modification, non-steroidal anti-inflammatory drugs (NSAIDs) and physical therapy focused on both core and scapular muscles strengthening (10). Patients can also undergo injection therapy before dealing with surgery. Among infiltrative therapies, several options are available including injection of corticosteroids, hyaluronic acid (HA), platelet-rich plasma (PRP), collagen and natural irritants (prolotherapy). However, these infiltrative therapies have shown variable and temporary effectiveness and many of them have no strong evidence (11). Furthermore, many trials evaluating the effectiveness of these infiltrative therapies use only clinical outcomes without image confirmations (11). As stated above, treatment of RC tendinopathies remains a big problem for clinicians, mainly because their pathogenesis is still largely misunderstood.

Collagen injections are used to treat different musculoskeletal disorders: they should stimulate tenocytes proliferation and migration by the synthesis of endogenous collagen and by the restoration of collagen fibers in damaged tendons (12). Dry Needling (DN) is one of the many options available for the management of tendinopathy. DN is especially recommended in the early stage of the disease because of its potential to provide pain relief causing minimal side effects (13).

The main hypothesis of this randomized controlled trial is that ultrasound-guided collagen injections (USG-CI) are superior to ultrasound-guided dry needling (USG-DN) in the management of pain and function in patients suffering from SSP chronic tendinopathy. For this aim, we evaluated whether collagen injections are superior to DN in reducing pain, improving function, and favoring tissue repair in patients with chronic SSP tendinopathy. To the best of our knowledge, this is the first randomized-clinical trial comparing these two procedures for the treatment of SSP tendinopathy.

## 2 Materials and methods

### 2.1 Study design

The current study is a randomized clinical trial that was performed from March 2024 to October 2024 at our Rehabilitation Centre.

### 2.2 Enrolment

**Figure 1:**
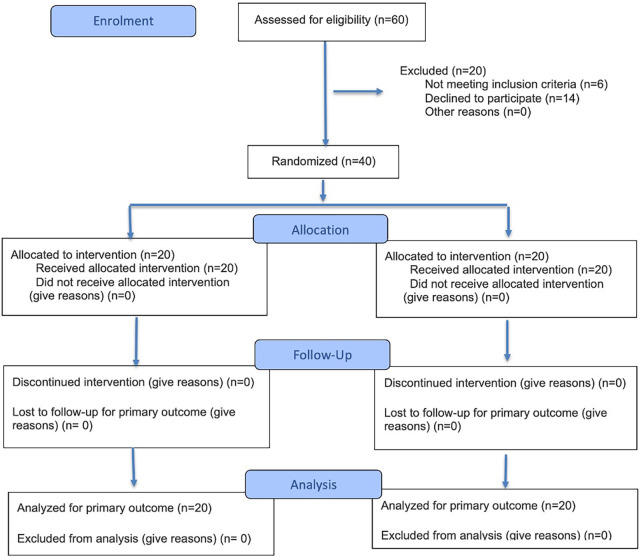
CONSORT flow diagram.

Forty patients suffering from SSP tendinopathy were recruited (Figure 1). All the outpatients who referred shoulder pain and disability were evaluated to verify the criteria for the enrollment and underwent ultrasound and x-ray.

### 2.3 Inclusion and exclusion criteria

The inclusion criteria were: age >18 years, symptomatic SSP tendinopathy of at least 6 months of duration, pain triggered by overhead activities, positive impingement sign (Neer test), pain with supraspinatus testing (Jobe's test), ultrasound evidence of SSP tendinopathy, and lack of injection therapy in the last 6 months. The exclusion criteria were previous shoulder surgery, RC tears greater than 50% of the tendon thickness, adhesive capsulitis, inflammatory arthritis, acromioclavicular joint pain, x-ray evidence of glenohumeral osteoarthritis, and previous fractures/bone tumors/osteonecrosis of the humerus. After a full and clear description of the study protocol, all patients enrolled were invited to sign the informed consent.

### 2.4 Outcomes

The primary outcome measure was the pain relief and functional improvement based on the Constant-Murley shoulder outcome Score (CMS) and the Disabilities of the Arm, Shoulder and Hand questionnaire (DASH).

The secondary outcome was the improvement of the tendon appearance at the ultrasound evaluation based on the Ingwersen score.

### 2.5 Ethical considerations

The study was carried out in accordance with the principles of the Declaration of Helsinki and approved by our Ethics Committee.

### 2.6 Randomization

Selected patients were divided into two groups using computer-generated random numbers: Group A (20 patients) underwent USG-CI while group B (20 patients) received USG-DN. Generation of random sequence was done by independent personnel not involved in the conduct of the study. The access to the random sequence was restricted to the investigators until assignments occur. The allocation remains blinded to outcome adjudicators and data collectors until the end of the study.

### 2.7 Interventions

**Figure 2:**
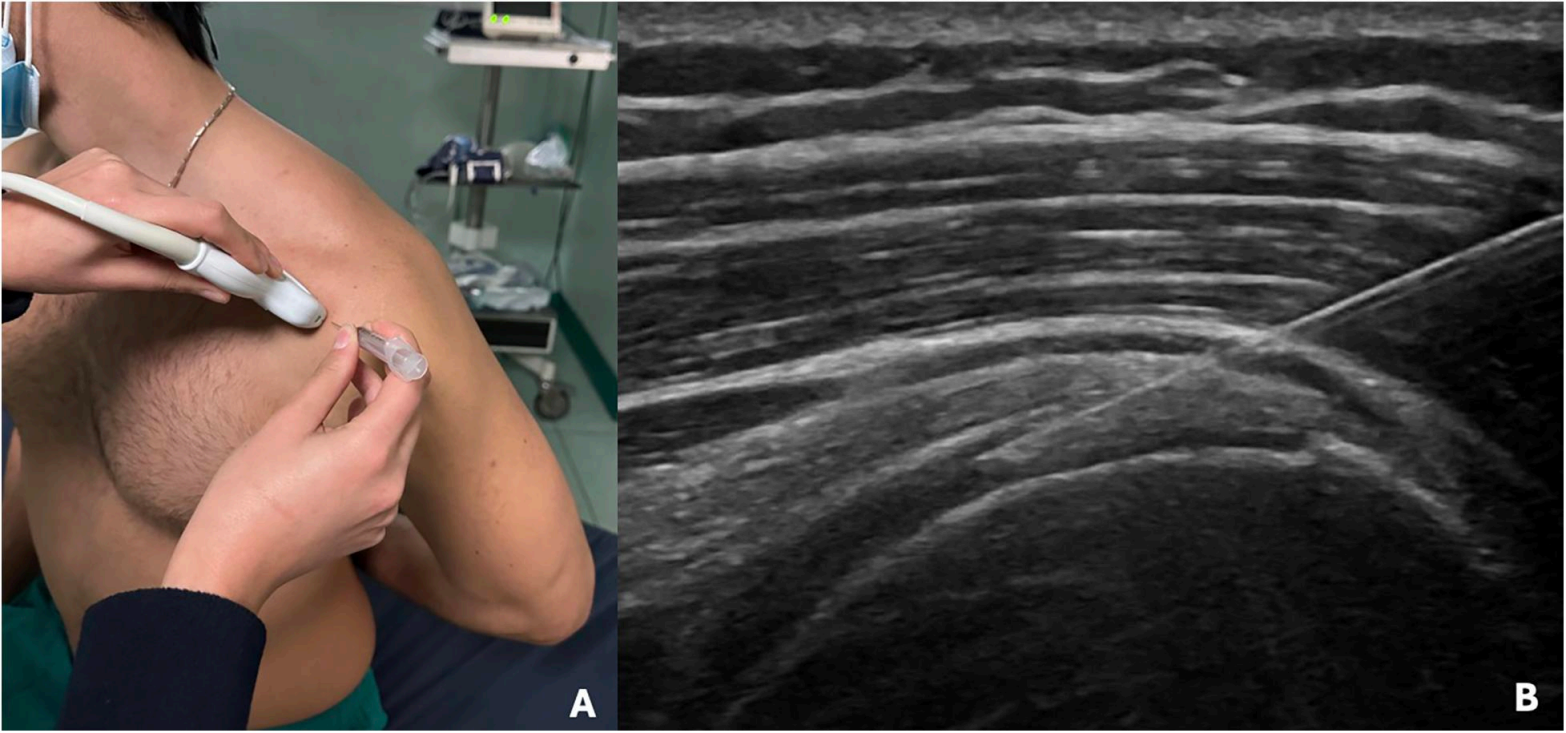
**(A)** Patient positioning and ultrasound probe orientation to perform the procedures. **(B)** Ultrasound image showing the needle into the SSP tendon.

**Figure 3:**
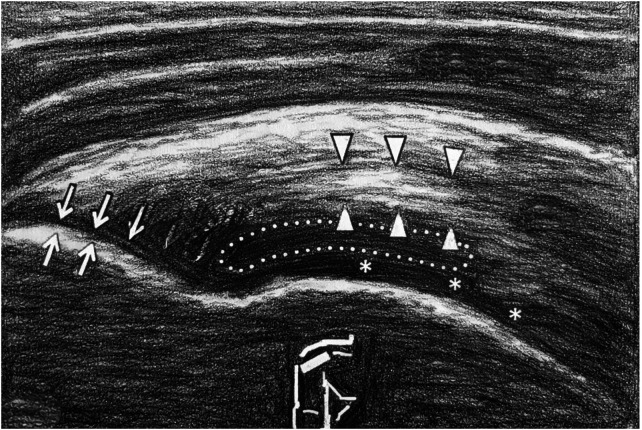
Ultrasound anatomy of the shoulder's anterior approach. ↑: SSP tendon insertion point on the greater tuberosity; ▴: typical hyperechogenic tendon area; *: humeral head cartilage.

CI were performed by the senior author (Figure 2). Injections were done using an anterior approach (Figure 3). Patient was seated on a chair with the arm in internal rotation to expose as much of the SSP tendon as possible. This position was best achieved by placing the patient's arm behind his back. The skin disinfection was performed with povidone iodine. A 25-gauge needle was directed towards the one or more hypoechoic areas of the SSP tendon as guided by ultrasound until the tip of the needle was seen in the correct position, and then the collagen was injected slowly. A 2 ml vial containing 10 mg onflow molecular weight hydrolyzed collagen peptides with added Vitamin C and Magnesium was administered. Only a little amount of the collagen was injected intra-tendinously due to the rapid raise of pressure at the tip of the needle. The remaining amount of the vial was injected peri-tendinously in withdrawing the needle. A high frequency linear transducer (8–15 MHz) was employed, keeping its long axis parallel to the long axis of the needle. Sterility was guaranteed by cleaning the area of interest with povidone iodine, by cleaning the transducer with disinfectant, and by covering the transducer with sterile cover after applying a layer of gel over the transducer. Patients were invited to keep the shoulder at rest until the day after. Each patient underwent a cycle of four USG-CI at weekly intervals. The senior author also performed all the percutaneous DN, using real- time ultrasound guidance with a sterile probe cover. The patient was lying on a bed with the arm in internal rotation. The skin disinfection was performed with povidone iodine. The 30-gauge needle was guided to the one or more hypoechoic areas of the tendon by an anterior approach and then it was passed through the tissue approximately 20/30 times. A high frequency linear transducer (8–15 MHz) was employed, keeping its long axis parallel to the long axis of the needle. Patients were invited to keep the shoulder at rest until the day after. Each patient underwent a cycle of two USG-DN at four weeks interval.

The same rehabilitation post-treatment protocol consisting of 12 sessions of physical therapy and proprioceptive training was carried out by patients of both group A and B (three sessions per week, starting one week after the last injection).

### 2.8 Measurement parameters

Outcomes' measurement was performed using CMS and DASH scores, and with an ultrasound evaluation using the ordinal grading scale by Ingwersen. The CMS is a multi-item functional scale assessing pain, activities of daily living (ADL), range of motion (ROM) and strength of the shoulder. CMS is a widely used scale in the assessment of shoulder function, and ranges from 0 to 100 points, representing worst and best shoulder function, respectively (14).

The DASH is a patient-reported outcome measure with 36 questions on coping in different everyday-life tasks. The scale ranges from 0 to 100, with a low number indicating better function (15).

The Ingwersen score is an ordinal grading scale used for the evaluation of subtle structural changes in rotator cuff tendons (Table 1) (16).

**Table 1:** Ultrasound assessment for grading structural tendon changes in supraspinatus tendinopathy by Ingwersen KG et al.

Grade	Description
0	Normal tendon
1	Thickening
2	Disruption without calcifications
3	Disruption with microcalcifications
4	Disruption with macrocalcifications
5	Partial rupture

All measurements were made at the enrolment (T0) and at 2-week (T1), 1-month (T2) and 3-month (T3) follow up, except for ultrasound evaluation that was only done at T0 and T3.

### 2.9 Statistical analysis

An independent observer who was unaware of the allocation and treatment arms of participants has collected primary and secondary outcomes. Data analysis was done using the S.P.S.S. version 26.0 statistical software. To incorporate the data of the participants into data analysis regardless of the protocol adherence, all the analyses were performed in accordance with the intention to-treat principle. Appropriate sample size calculation and power analysis were assessed during the planning of the clinical study. To detect a difference of 20% (Effect size) between groups with a power of 80% and a significance level of 0.05, a required a-priori sample size of 20 patients per group was calculated using G*Power software (ver. 3.1.9.7; Heinrich-Heine-Universität Düsseldorf, Düsseldorf, Germany), assuming a drop-out rate of 5%. Data were expressed as frequencies and percentages for categorical variables and mean and standard deviation (M ± SD) for continuous variables. Intergroup comparison of CMS and DASH outcomes was analyzed using repeated measurement A.N.O.V.A. Ultrasound assessments sec. Ingwersen et al. were compared using medians and interquartile ranges (IQR). Statistical significance was defined as a *p*-value of <0.05.

## 3 Results

Baseline demographic and clinical data of enrolled patients are shown in Table 2. The two groups were homogeneous in terms of age, sex and BMI.

**Table 2:** Baseline demographic and clinical characteristics for each group.

Demographic variables	CI group (*n* = 20)	DN group (*n* = 20)
Sex (M/F)	12/8	8/12
Age (years)	38–78 (53.9)	41–78 (57.3)
BMI (18.5–30)	22.5–27 (23)	20–25.5 (22.5)
CMS	27–85 (53.7)	39–60 (51)
DASH	4–66 (37.35)	11–66 (40.5)

We performed intra- and inter-group variance analysis to compare the response of the patients with chronic SSP tendinopathy to the two different treatment modalities. At the 2-week post-injection evaluation, no statistically significant differences were observed between the two groups regarding both CMS and DASH. The mean CMS was 63.20 (±10.28) points for USG-CI group and 63.85 (±9.93) for the USG-DN group. The between-group difference was 0.65 points (*p* = 0.839). As regards DASH, the mean score was 28.75 (±14.35) points for USG-CI group and 28.45 (±11.09) for the USG-DN group. The between-group difference was 0.3 points (*p* = 0.941).

The mean CMS at 1 month was 72.85 (±10.19) points for USG-CI group and 64.20 (±13.78) for the USG-DN group. The between-group difference was 8.65 points (*p* = 0.029). As regards DASH, the mean score at 1 month was 19.45 (±9.75) points for USG-CI group and 33.6 (±25.09) for the USG-DN group. The between-group difference was 14.15 points (*p* = 0.024).

Three months after the last injection, we observed a mean CMS of 76 (±13.51) points for USG-CI group and 63.1 (±20.71) for the USG-DN group. The mean DASH was 18.85 (±12.87) points for USG-CI group and 33.84 (±27.88) for USG-DN group. The between-group differences were 12.9 and 14.99 points respectively (*p* = 0.025 and 0.045).

**Figure 4:**
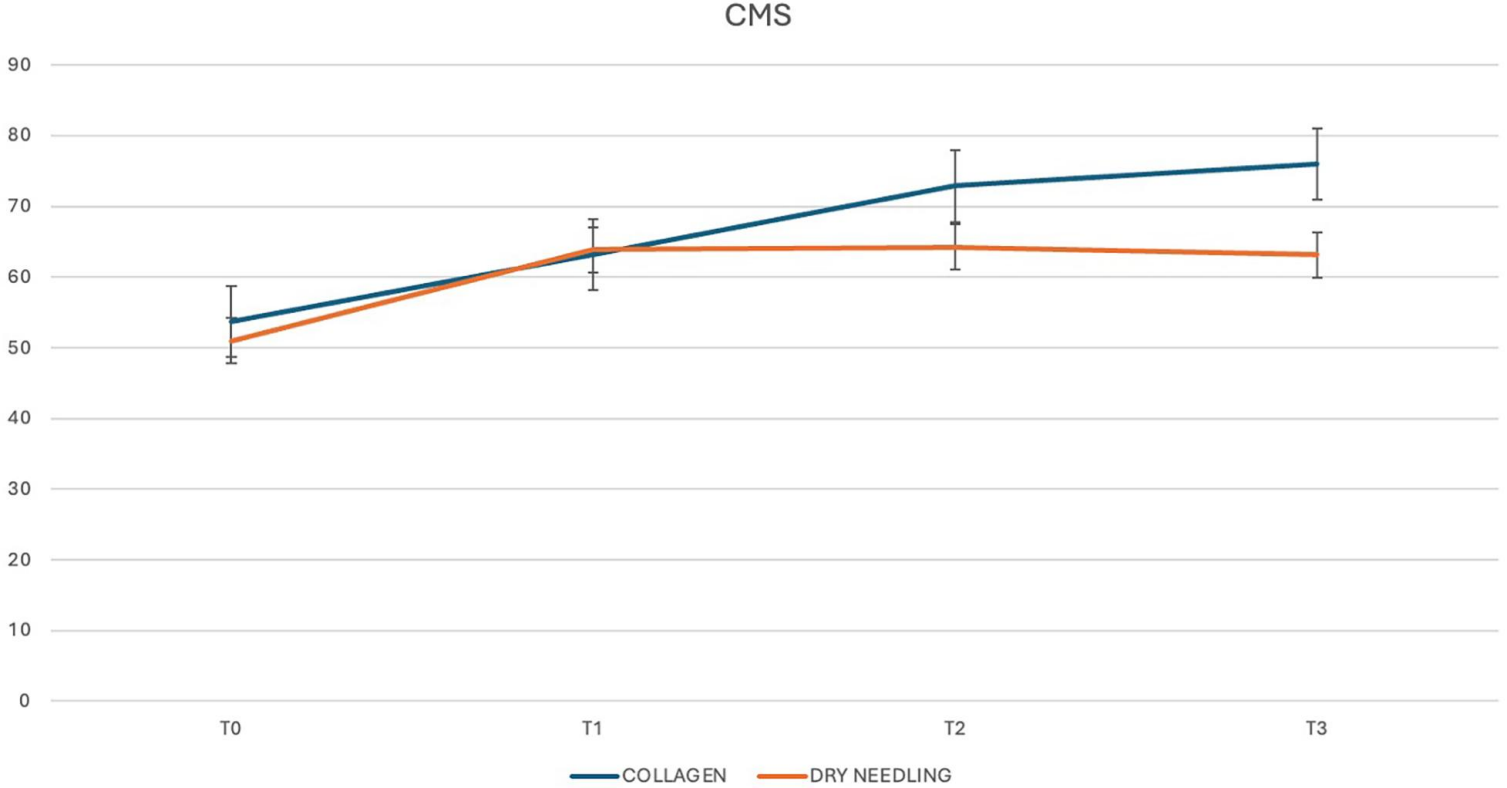
CMS score trend through the different follow-ups.

**Figure 5:**
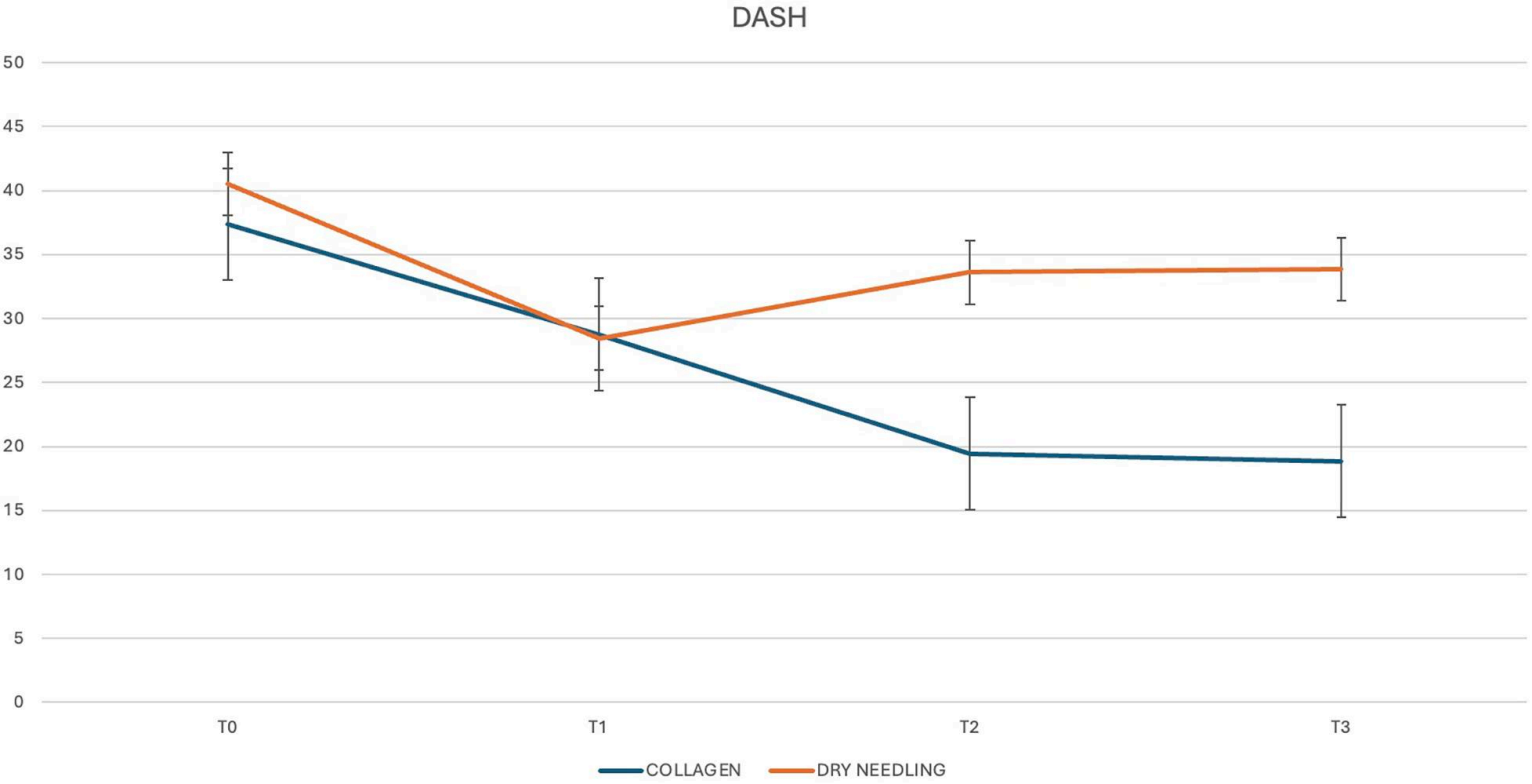
DASH score trend through the different follow-ups.

CMS and DASH trends trough the different follow-ups are shown in Figures 4, 5.

A radiologist with over 10 years of experience in musculoskeletal ultrasound, unaware of group allocation, assigned a numerical score to patients' SSP tendons at the enrolment and at 3-month follow-up, according to Ingwersen scale. At T0 the median score was 3 (IQR: 1.75) for USG-CI group and 2.5 (IQR: 1.25) for the USG-DN group. Three months after the last injection, the radiologist assigned a median score of 2 (IQR: 1.5) to USG-CI patients and 2.5 (IQR: 1.25) to USG- DN patients. No adverse events were recorded in both groups.

## 4 Discussion

In our study, both USG-CI and USG-DN improved pain and functionality at each follow-up in patients affected by chronic SSP tendinopathy. However, the inter-group analysis showed a statistically significant improvement in the USG-CI group for CMS and DASH scores, and a greater improvement in SSP morphology at three months.

The reason for the superiority of collagen to DN may be that USG-CI induce regenerative pathways by the activation of the integrin receptors in fibroblast cell membranes (17, 18); consequently, the growth factor cascade initiates the synthesis of endogenous collagen (19), finally healing the damaged collagen fibers and leading to proper alignment (20–22).

Although less effective than collagen, DN itself might have a therapeutic effect on SSP tendinopathy. This could be explained by the fact that DN may induce bleeding, which in turn releases certain growth factors that stimulate the healing process (23).

Among conservative treatments, different kind of injections have been proposed in the last years for the treatment of chronic SSP tendinopathy, but they have achieved controversial effectiveness according to the scientific literature (11, 24, 25). In this context, collagen injections may represent an effective therapeutic option. However, the quality of the current available on its use is low, with only one level-I study, one level-III and four level-IV studies published so far, preventing definitive recommendations regarding the indication for the use of collagen for chronic SSP tendinopathy or, more generally, for RC tendinopathy (including partial-thickness tears) (18, 26–30). The use of DN for SSP tendinopathy or more generally for RC diseases has been less studied, with only one level-I study and two level IV studies published so far of which one used DN coupled with electrical stimulation (23, 31, 32).

Regarding the use of collagen for SSP tendinopathy or, more generally, for RC tendinopathy, four studies showed both clinical and radiological improvements for RC partial-thickness tears and RC tendinopathy with the use of collagen, while non-significant changes were found only in one article on RC partial-thickness tears (26–30). When patients treated with collagen injections were compared to non-treated patients, outcomes were significantly better (27, 33).

In their 2018 study, Randelli et al. found that an injectable medical device (MD) containing type I collagen of swin derivation induced an anabolic phenotype if it was added to a plate with cultured human tenocytes. The injected MD promoted collagen type I secretion and increased mRNA levels of TIMP (an inhibitor of matrix metalloproteinases-1, the protein most responsible for collagen degradation), and so favored tendon homeostasis and repair (12). In a successive paper, Randelli and collaborators confirmed that collagen-based MD, added to cultured human tenocytes, affects collagen turnover favoring cell migration, and showed that the induced effect represented a mechanical input (34). Following these *in vitro* results, collagen injections have been recently proposed for the treatment of different musculoskeletal disorders such as tendinopathies and osteoarthritis (35–37).

Corrado et al. treated 18 patients with SSP tendinopathy with the same collagen used for this study and at the same interval (four U.S.-guided peri- and intra-tendineous injections, once a week, of type I collagen) and evaluated them at the same follow-ups (two weeks, one and three months). Improvements of CMS and DASH scores were obtained from baseline (53.11 ± 12.7 and 37.72 ± 19, respectively) to the last follow-up at three months (75 ± 12.9 and 18.67 ± 13, respectively), as well as of the ultrasound grading score examination that are comparable with the results of the current study (38).

Buda et al. conducted a multicentric, observational prospective study on 71 patients diagnosed with RC tendinopathy and treated with two subacromial injections of a 4 mg/2 ml collagen at 13 ± 2.9 days apart. Their results showed, among the other considered scores, a gradual improvement of the mean CMS from 63.76 (at baseline) to 80.81 at one month and to 84.07 at the last follow-up (six months) with a trend that was similar to the one retrieved from our results (increasing CMS score among the follow-ups) (29).

These favorable outcomes were also observed in two case-reports on the use of injectable collagen for RC partial-thickness tears in which a complete healing of the tendon tear at the last follow-up, along with improvements in shoulder pain and function, was reported (18, 36). Therefore, the use of collagen injections for RC tendinopathy, including partial-thickness tears, appears to be reasonable, even if the quality of the studies is relatively low, so several questions still need to be addressed (29).

DN is another reliable option for the treatment of tendinopathy as reported by Nuhmani et al. in his systematic review. Nuhmani et al. investigated the effectiveness of DN in the management of tendinopathies. Their results suggested that DN appears to be as effective as other treatment methods in relieving pain and other associated symptoms immediately and up to 6 months (short term and medium-term) (39). In DN, injection may induce bleeding and convert the chronic degenerative process of tendinopathy into an acute process by releasing growth factors that induce the inflammatory process and stimulate healing (40).

Rha et al. compared DN and PRP for patients with SSP tendinopathy using a standard protocol for the USG-DN treatment (two procedures at four-week intervals with the needle passed through the lesion of the tendon approximately 40–50 times) that was very similar to the one adopted in the current study (23). Their findings showed that both PRP injections and DN demonstrated therapeutic effects on SSP tendinopathy, but PRP injections provided more symptomatic relief and functional improvement than DN at six-month follow-up.

However, improvement in the range of motion of the shoulder were not different between the PRP and DN groups, with the latter showing unexpectedly good results in some patients. Furthermore, the ultrasound evaluation showed improvements of the SSP tendon structure at six months only in the PRP group.

In the case-report published by Settergren, a single session of DN for SSP tendinopathy with the tendon being fenestrated approximately 15 times was sufficient to provide a full resolution of symptoms and improvement in the SSP structure at the ultrasound examination ten days after the procedure (32).

Despite the good outcomes reported with the use of DN, its use for SSP tendinopathy remains questionable due to the very low available scientific evidence. For this reason, other types of injections may be preferred according to two recent systematic review about the efficacy of injections for RC tendinopathy and partial-thickness tears (24, 25).

The main strength of the present study is that, to the best of our knowledge, this is the first randomized controlled trial comparing collagen injections and DN for the treatment of SSP tendinopathy, providing further evidence especially on the efficacy of collagen for the treatment of this condition. As recently demonstrated by Dinc et al., collagenic injections leads to lower levels of pro-inflammatory cytokines, interleukin-1 beta (IL-1β), interleukin-6 (IL-6), and tumor necrosis factor- alpha (TNF-α), as well as cartilage degradation markers, matrix metalloproteinase-13 (MMP-13), C-terminal crosslinked telopeptide of type II collagen (CTX-II), and cartilage oligomeric matrix protein (COMP). Additionally, oxidative stress indicators including inducible nitric oxide synthase (iNOS), total oxidant status (TOS), and oxidative stress index (OSI) are decreased (41). What has been reported is confirmed by two recent clinical studies. Tassinari et al. reported faster recovery after hip arthroscopy, if a post-operative injection of hydrolized collagen peptides has been administered (42). In a similar way, Latini et al. reported good clinical outcomes after hydrolized collagen peptides to treat partial SSP tendon tear (30). Laboratory studies have in fact shown that maintaining the collagen triple-helix and cross-links between the molecules is essential for the elastic energy storage in tendons, which involves direct stretching, preventing injuries and recurrences (43, 44). On the other hand, collagen injections need to be administered more frequently than DN. In fact, as report by Nuhmani et al. in his metanalysis (39), there is consensus that DN should be performed at least four weeks apart, as it is more aggressive on the tendon and causes bleeding, which ultimately determines the beneficial effect. Performing it more frequently would not add benefits, but rather would increase the risk of adverse events and patient's disconfort.

Furthermore, the absence of follow-up loss meant that patients' compliance to treatment was satisfactory. Finally, we performed ultrasounds to diagnose the tendon pathology, to identify the most appropriate injection site, and to guide the needle during the injection.

The limitations of the current study are the relatively small sample of patients (20 in each group) and the short duration of follow-ups (three months). Future research directions may also be highlighted.

The goal is also to tailor the treatment as closely as possible to the characteristics of each patient. The effectiveness of this treatment could also be studied in particular groups of patients, such as those affected by tendinopathy secondary to antegrade humeral nailing (45). Collagen has certainly proven to be a superior treatment option in our study, but, in the future, we might consider refining the proposed treatment based on the MRI scans tendinopathy's characteristics to achieve better results for a patient-specific approach.

In addition, it would be interesting to investigate in a pre-clinical or histological study if the effects of USG-CI in treating tendinopathy are due to collagen itself more than to bleeding caused by needle, underlying the superiority of the collagen over DN.

## 5 Conclusions

According to our randomized controlled study, USG-CI provided more significant pain relief, functional and tendon structure improvements in patients with SSP tendinopathy when compared to DN. These findings suggested that USG-CI could be a feasible, safe, easy to administer and relatively cheap therapeutic option for SSP tendinopathy, including partial- thickness tears. Further studies are required to evaluate the benefits of collagen in bigger RC tears and to confirm the effects of collagen on radiological and histological changes of damaged tendons.

## Data Availability

The raw data supporting the conclusions of this article will be made available by the authors, without undue reservation.
